# Validity of bioelectrical impedance analysis in predicting total body water and adiposity among Senegalese school-aged children

**DOI:** 10.1371/journal.pone.0204486

**Published:** 2018-10-11

**Authors:** Adama Diouf, Ousmane Diongue, Mégné Nde, Nicole Idohou-Dossou, Mbeugué Thiam, Salimata Wade

**Affiliations:** Laboratoire de Nutrition, Département de Biologie Animale, Faculté des Sciences et Techniques, Université Cheikh Anta Diop (UCAD) de Dakar, Sénégal; University of Buea Faculty of Health Sciences, CAMEROON

## Abstract

**Introduction:**

Childhood obesity is currently a serious public health challenge in developing countries. Therefore, an accurate assessment of adiposity is required. The objective of this study was to validate BIA prediction equations for the assessment of total body water and adiposity or percentage of body fat for the first time in Senegalese school-aged children.

**Methods:**

One-hundred-fifty-one (151) pupils who were 8–11 years old were randomly selected from four public schools in Dakar. The body composition measured by deuterium dilution method (DDM) was used as the reference method and compared to that predicted by BIA using a multi-frequency analyser. Stepwise backward multiple linear regression was performed to calculate TBW and %BF in a subsample, which were then validated in the rest of the sample. The Bland and Altman approach was used to assess the agreement between the two methods (bias and limits of agreement).

**Results:**

FFM was higher in boys (24.6±6.9 kg) compared to girls (21.2±3.3 kg; P<0.001), and FM was lower in boys: 3.7 kg [0.9–26.4] compared to girls: 4.5 kg [1.7–22.7]. Overall, 11.3% of children presented excess adiposity (%BF >25% in boys, and >30% in girls) and 2.0% were obese according to WHO cut points for obesity (BMI z-score >+2.0). The equations developed were as follows: TBW = 0.376(Height^2^/Z_50_)-0.470 (sex) +0.076(weight) +0.065(height)-2.28.

%BF = -1.10(height^2^/Z_50_) +3.14(sex)+1.57(weight)-4.347. These specific equations showed good precision and a low and non-significant mean bias (0.11 kg, P = 0.279; and 0.19 kg, P = 0.764) for TBW and %BF, respectively.

**Conclusion:**

The newly developed equations can be used as an accurate and alternative screening tool for the assessment of obesity among children in various settings.

## Introduction

Despite the substantial economic growth observed in recent decades, low and middle-income countries (LMICs) are still facing a high prevalence of undernutrition and the growing problem of obesity and related diseases [1].

Childhood obesity is a serious public health challenge. Therefore, accurate and reliable indicators are required for early detection or diagnosis of adiposity excess and trends of child obesity in developing countries. Anthropometric measurements are widely used in epidemiological studies and clinical screening. Body mass index (BMI) is recommended by WHO for the assessment of adiposity in children and adolescents [2]. However, as reported in the literature, BMI may not reflect the various body compartments as it is a global proxy that measure both fat free mass (FFM) and fat mass (FM), the two major body compartments. Body composition is an indicator that reflects the relative proportion of these two compartments. The amount and distribution of FFM and FM change with age and are important health outcomes through the life cycle. The accuracy of BMI differs substantially according to the degree of body fat [3–4]. Furthermore, due to the significant physiological changes that occur during childhood, FFM and FM should be considered when assessing the nutritional status of children in terms of how overweight or obese the child is and the related health risks. Indeed, an increased proportion of fat mass and decreased proportion of FFM during this period were reported to be associated with an increased risk of developing chronic disease in later life [5]. Body composition in children has commonly been assessed using reference methods, including densitometry, X-ray two-photon absorption, Dual-energy X-ray absorptiometry (DEXA), total potassium and the stable isotope dilution method. Isotopic dilution is one of the gold standard methods that precisely measures the dilution space of deuterium or oxygen-18 from which TBW and other body compartments (fat free mass and fat mass) are calculated. The deuterium dilution method (DDM) has been used both in adults and children [6–7]. Unfortunately, although accurate and relatively simple, this technique is onerous in large epidemiological studies. A simpler field technique, such as bioelectrical impedance (BIA), can be an alternative to estimate body composition. BIA is non-invasive, relatively inexpensive, easy to use and suitable for larger studies and has widespread applications in the field of nutrition [7–9]. BIA measures the resistance of the current that is inversely proportional to the amount of TBW, the conductive material.

Numerous population-based BIA studies have been conducted to develop TBW and FFM prediction equations using DDM as the reference method. Nevertheless, to the best of our knowledge, few BIA equations have been validated for the prediction of %BF among children, particularly those from Sub-Saharan Africa [10–11]. Moreover, both TBW and %BF BIA prediction equations, mostly developed in Caucasians, are population-specific and may be inappropriate in African populations [12–16]. Hence, the objective of the present study was for the first time to develop and validate BIA prediction equations as an alternative tool for the assessment of TBW and adiposity among Senegalese school-aged children using DDM as the reference method.

## Materials and methods

### Study design and subjects

The present cross-sectional validation study was conducted from December 2–19, 2013, in an urban area of Dakar. The study used a two-stage random sampling procedure: in the first stage, four elementary public schools were randomly selected from the 149 schools of the academic institutions of Dakar. Second, a sample of 151 prepubescent school-aged children (74 boys, 77 girls) aged 8–11 years was recruited within the selected schools. The inclusion criteria were children in good general health and consent from the children and parents. The exclusion criteria were presence of physical or mental disabilities. The Ministry of Education authorized the study approved by the ethical committee of University Cheikh Anta Diop of Dakar (0011/CER/UCAD). The study objectives and testing procedures were explained to both the pedagogical team, the parents, and the children. But as the children were minors, a signed consent form was obtained from parents before starting data collection.

### Anthropometry

Anthropometric measurements were performed using standardized procedures. Body weight was measured to the nearest 50 g using electronic scales and without excess outer clothing (SECA 869, GmbH & Co, Hamburg, Germany), and height was measured to the nearest 0.5 cm with a height/length SECA 217 stadiometer (SECA, California, USA). Height-for-age z-scores (HAZ) and BMI-for-age (BMI z-score) were calculated, and children were classified as underweight, normal weight, overweight, and obese using the WHO growth standard reference for children and youth 5–19 years of age [2].

### Body composition assessment

#### Bioelectrical impedance analysis (BIA)

BIA was measured with a multi-frequency analyser (Xitron 4000B. Xitron Technologies. California. USA). Two pairs of electrodes were placed on the hand, wrist, foot and ankle on the left side of the body following a standardized procedure. Prior to measurements, subjects voided their bladder and were then rested in a supine position with their arms and thighs held apart for 15 minutes. The same investigator performed all of the measurements. The accuracy of the instrument was tested by using a 422 Ω standard resistor purchased with the analyser. The impedance (Z) values provided by the device were reported to the nearest 0.1 Ω from a digital display. From the spectrum of 50 measurements (5 to 1000 kHz), the impedance at 50 kHz was used in the present study. The impedance index was calculated by height squared divided by impedance (height^2^/Z), as this is a crude approximation to the body volume and should be highly correlated with a laboratory estimate of TBW [8–9, 11].

#### Deuterium oxide dilution method

An accurately weighed dose (0.5 g/kg body weight) of deuterium oxide (99.8% purity, Cambridge Isotope Laboratories Inc. Andover, MA) was orally administered to the children followed by 50 mL of local tap water [6]. Prior to dosing, the children provided a pre-dose saliva sample (4 mL) into a clean, sterile and dry tube to determine the background or natural deuterium enrichment. The post-dose samples were collected 3 hours after dosing. Saliva samples were stored at −20 °C until analysis. The deuterium enrichment in the saliva was measured using a Fourier Transform Infrared Spectrometer (FTIR IR-Affinity, Shimadzu, Nakagyo-ku Kyoto, Japan) as describe elsewhere [6]. Laboratory quality control of the FTIR was performed to assess the basic performance of the instrument using a polystyrene film. The accuracy of the measurements was checked using eight individually gravimetrically prepared standardsAn internal pool of saliva sample was also prepared and analysed prior to sample measurements. All of the saliva samples were measured in triplicate, and enrichments (mg/kg or ppm) were obtained with the isotope software. TBW was calculated from the deuterium oxide dilution space using a correction factor of ~4% of deuterium exchange with the non-aqueous compartment of the body [17]. FFM was determined considering the hydration factor, which varied according to age and gender among children [18]. BF and %BF were calculated as the difference between body weight and FFM in relation to body weight. Excess adiposity was defined as %BF >25% in boys and >30% in girls [19].

### Statistical analysis

Data entry and quality control were performed using EpiData version 3.1 (the EpiData Association Odense. Danemark), Microsoft Excel 2007 (Microsoft corporation Redmond. USA) and WHO AnthroPlus version 1.0.4 (OMS 2007). Statistical analyses were carried out by STATA/SE version 11.0 (STATA Corporation, Texas, USA). Data were expressed as the mean ± SD and percentage. The baseline characteristics were compared using Student’s t-test. TBW and %BF prediction equations were developed by using stepwise backward multiple regression analyses. The total sample was split into two groups: prediction and cross-validation groups. The TBW prediction equation was developed using TBW from DDM as a dependent variable and the impedance index (height^2^/Z_50_) as well as age, sex (male = 1, female = 2), height and weight as predictor variables. The %BF prediction equation was also derived from the prediction group with %BF measured by DDM as the dependent variable and impedance index (height^2^/Z_50_), age, sex, weight and height as independent variables. All of the conditions in the multiple regression analysis were tested (*i*.*e*., assumptions about the linearity of the model, normally distributed residuals and homoscedasticity). Outliers, defined as cases with standardized residuals < -3 and > +3, were excluded. Multicollinearity was evaluated using the variance inflation factor (VIF). A variance inflation factor <10 was considered appropriate [20]. A high R-square (R^2^) value and small root mean square error (RMSE) indicated the optimal model. For cross-validation purposes, the pure error (accuracy) was used to assess the performance of the prediction equation. Pure error was calculated as the square root of the sum of squared differences between the observed and the predicted values divided by the number of subjects in the cross-validation sample. The smaller the pure error, the greater the accuracy of the equation. The relationship between the measured and predicted values of TBW or %BF was assessed using Pearson’s correlation coefficient. The difference between the measured and predicted values (bias) of TBW and %BF was tested against zero (paired t test). Bland-Altman plots with 95% limits of agreement (± 1.96 x SD) were used to assess the concordance between the two methods [21]. For all analyses, a P value < 0.05 was considered statistically significant.

## Results

### Characteristics of the children

Descriptive statistics of the children by sex and group (prediction and validation) are shown in Table 1. One-hundred-fifty-one children (74 boys and 77 girls) with a mean age of 9.6 ± 1.0 years were included in the study. The mean (± SD) body weight, height, BMI and HAZ were 28.2 ± 6.5, 137.2 ± 7.8, -1.34 ± 1.20 and -0.19 ± 1.07, respectively, with 3 children suffering from stunting (HAZ < -2 z-score). Overall, there were no significant differences in anthropometric variables between boys and girls. Boys had a significantly greater FFM (24.6±6.9 kg vs 21.2±3.3 kg, P<0.001) and lower FM: 3.7 kg [0.9–26.4] vs. 4.5 kg [1.7–22.7]; *P<0*.*017*) than girls (*P<0*.*05*), and 11.2% (n = 17) of children had excess adiposity (%BF >25% in boys, and >30% in girls). The BMI-z score showed 2.0% prevalence of obesity (BMI >+2.0). The anthropometrics and body composition parameters were comparable in the prediction and validation groups.

**Table 1:** Baseline characteristics of the children in the prediction and cross-validation samples.

	Total sample (n = 151)	Prediction sample (n = 76)	Validation sample (n = 75)	*P*^1^
*Anthropometry*				
Boys % (n)	49% (74)	53% (39)	47% (34)	0.592
Age (years)	9.6 ± 1.0	9.5 ± 0.9	9.6 ± 1.1	0.725
Weight (kg)	28.2 ± 6.5	28.2 ± 6.6	28.3 ± 6.5	0.991
Height (cm)	137.2 ± 7.8	137.6 ± 7.4	136.8 ± 8.5	0.193
BMI z-score	-1.34 ± 1.20	-1.32 ± 1.20	-1.42 ± 1.18	0.592
HAZ	-0.19 ± 1.07	-0.07 ± 1.04	-0.35 ± 1.09	0.113
*Body composition*				
TBW (kg)	17.2 ± 2.7	17.2 ± 2.3	17.23 ± 3.0	0.914
FFM (kg)	22.8 ± 5.7	23.1 ± 6.9	22.5 ± 4.0	0.463
FM (kg)	4.4 [0.9–26.4]	4.1 [0.9–26.4]	4.5 [1.5–18.3]	0.533
%BF	18.7 ± 9.7	18.8 ± 11.3	18.6 ± 7.8	0.780
* Normal* % (n)	88.7 (134)	86.8 (66)	90.7 (68)	
* Obese* % (n)^2^	11.2 (17)	13.2 (10)	9.3 (7)	
BIA				
Impedance (Ω)	784.3 ± 85.6	785.0 ± 91.4	781.4 ± 82.0	0.760
Height^2^/Z_50_ (m^2^/Ω)	24.1 ± 4.3	24.2 ± 4.1	24.1± 4.6	0.844

FM (kg): expressed as median and interquartile range.

^1^*P*: *student t-test* (prediction vs. validation sample)

^2^Obese: %BF >25% in boys, %BF >30% in girls

*Height*^*2*^*/Z*_*50*_ = *Impedance index*

### Development of BIA prediction equations

**Fig 1:**
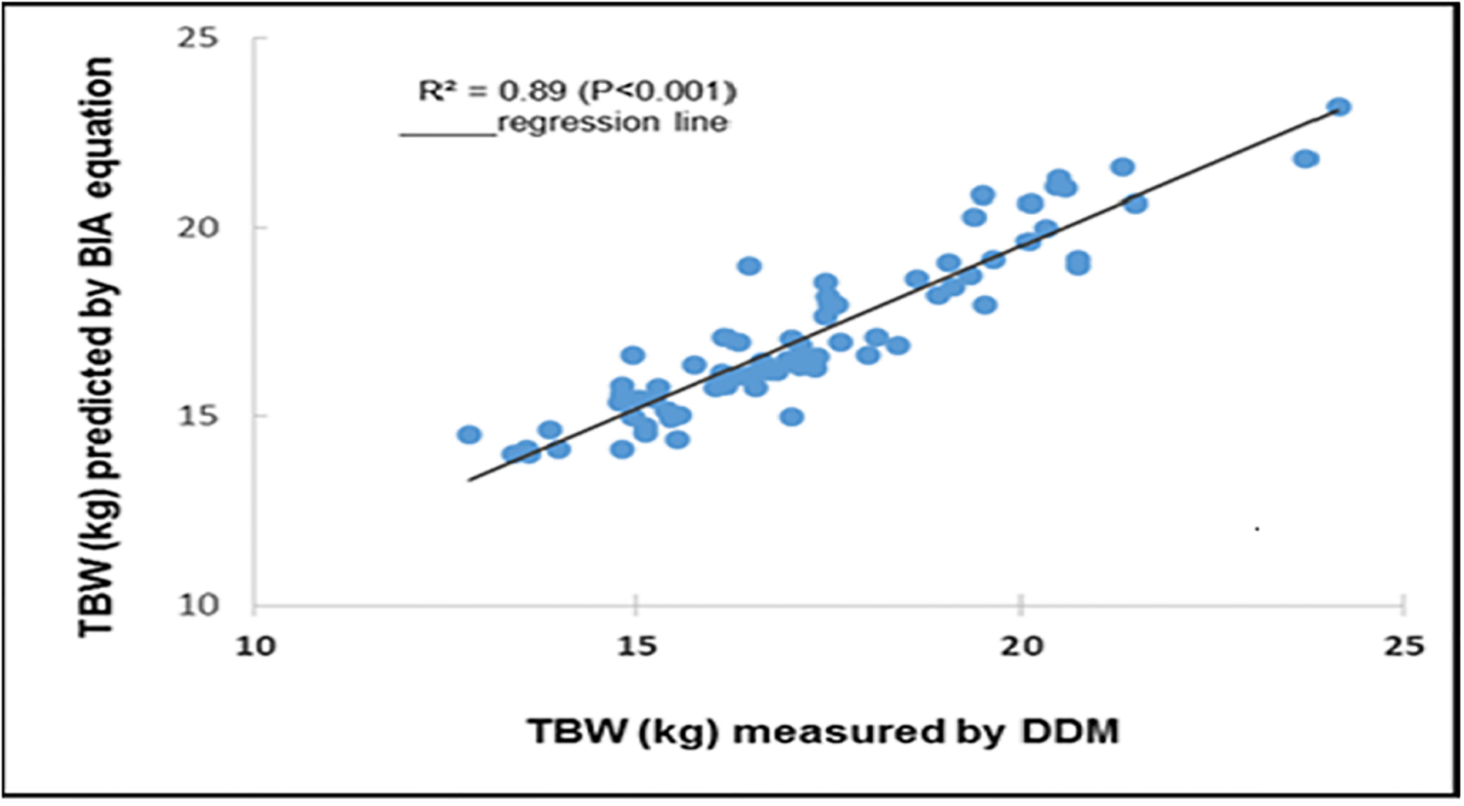
Correlations plot between TBW values from DDM and BIA were performed and r value measure the degree of linear connection between two variables.

**Fig 2:**
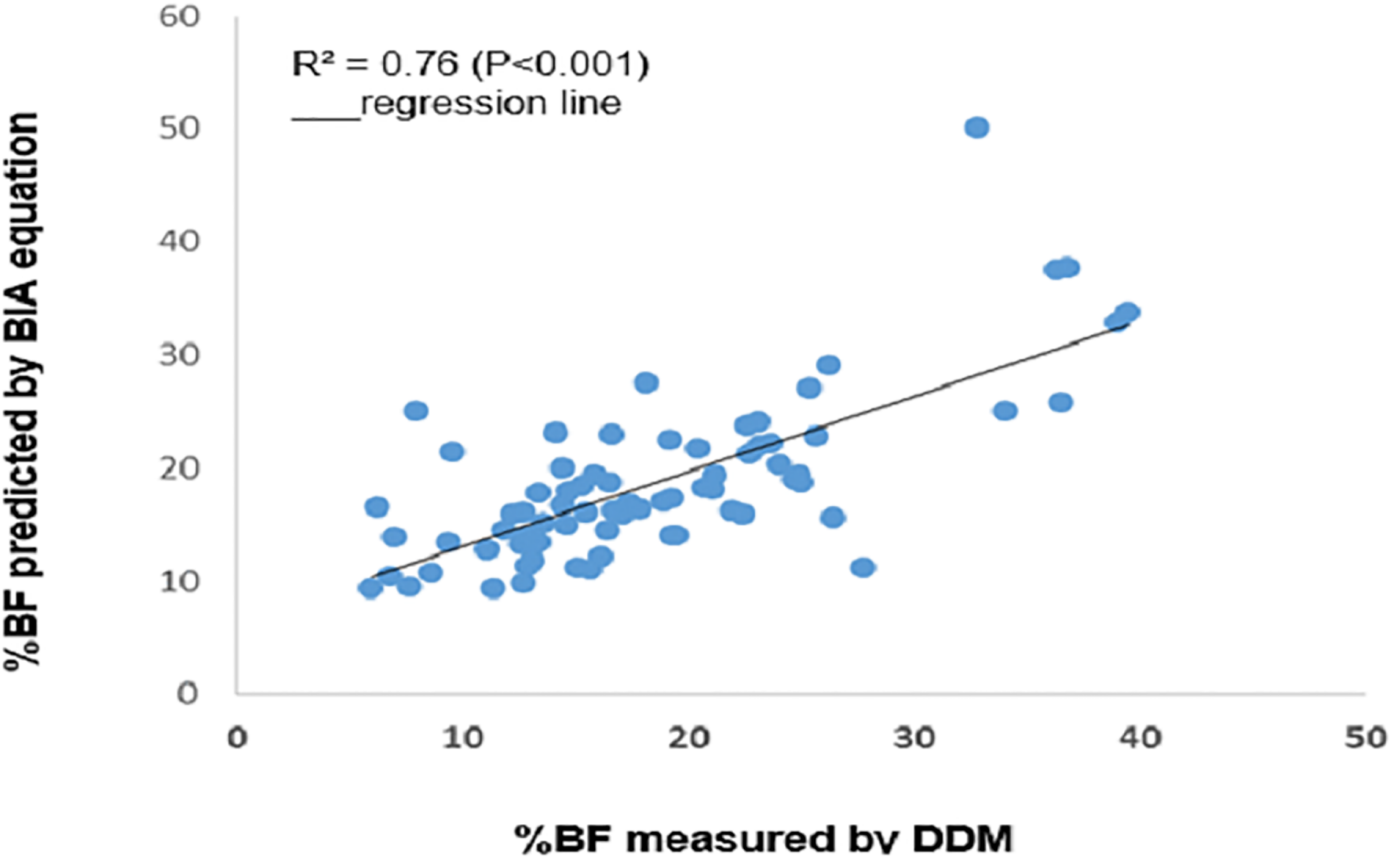
Correlations between %BF values from DDM and BIA were performed and r value measure the degree of linear connection between two variables.

A significant correlation was observed between TBW and % BF values measured by DDM and those estimated by the prediction equations (TBW: r = 0.90 p <0.001,% BF: r = 0.74, p <0.001) (Figs 1 and 2). In the final model, four variables (height^2^/Z_50_, sex, weight and height) were found to be significantly associated with TBW measured by DDM and accounted for the largest R^2^ (89.0%) and lowest RMSE (0.89 kg) (Fig 1). Three variables (height^2^/Z_50_, sex and weight) were significantly associated with %BF and accounted for 76% of its variation with a RMSE of 5.5% (Fig 2).

Age was not a significant predictor in any of the models. The final models did not show multicollinearity for both TBW and the %BF equation (no associations between independent variables) as determined by variance inflation numbers of 2.67 and 2.08, respectively (Table 2).

**Table 2:** BIA prediction equations for TBW and %BF.

Equations	R^2^	RMSE	VIF	Bias	Accuracy	Limits of agreement
TBW (kg) = 0.376(height^2^/Z_50_) - 0.470(sex) + 0.076(weight) + 0.065(height) - 2.28	0.89	0.89 kg	2.65	0.11 kg	0.96 kg	-1.67–1.89 kg
%BF = -1.104(height^2^/Z_50)_ + 3.136(sex) + 1.57(weight) - 4.347	0.76	4.5%	2.20	0.19%	5.6%	-10.78–11.17%

*RMSE*: *Root Mean Square Error; VIF*: *Variance Inflation Factor*

*Height*^*2*^*/Z50 = Impedance index*

Sex code; male = 1 and female = 2

### Cross-validation

The TBW and %BF equations developed in the prediction group were tested in the cross-validation group to assess their accuracy. The TBW prediction equation yielded an accuracy of 0.96 kg, and the prediction equation of %BF yielded an accuracy of 5.6%. The TBW and %BF values measured with DDM were highly correlated with the predicted values of TBW (r = 0.89; P<0.001) and %BF (r = 0.76; P<0.001). The new equations that were developed and their characteristics are shown in Table 2.

**Fig 3:**
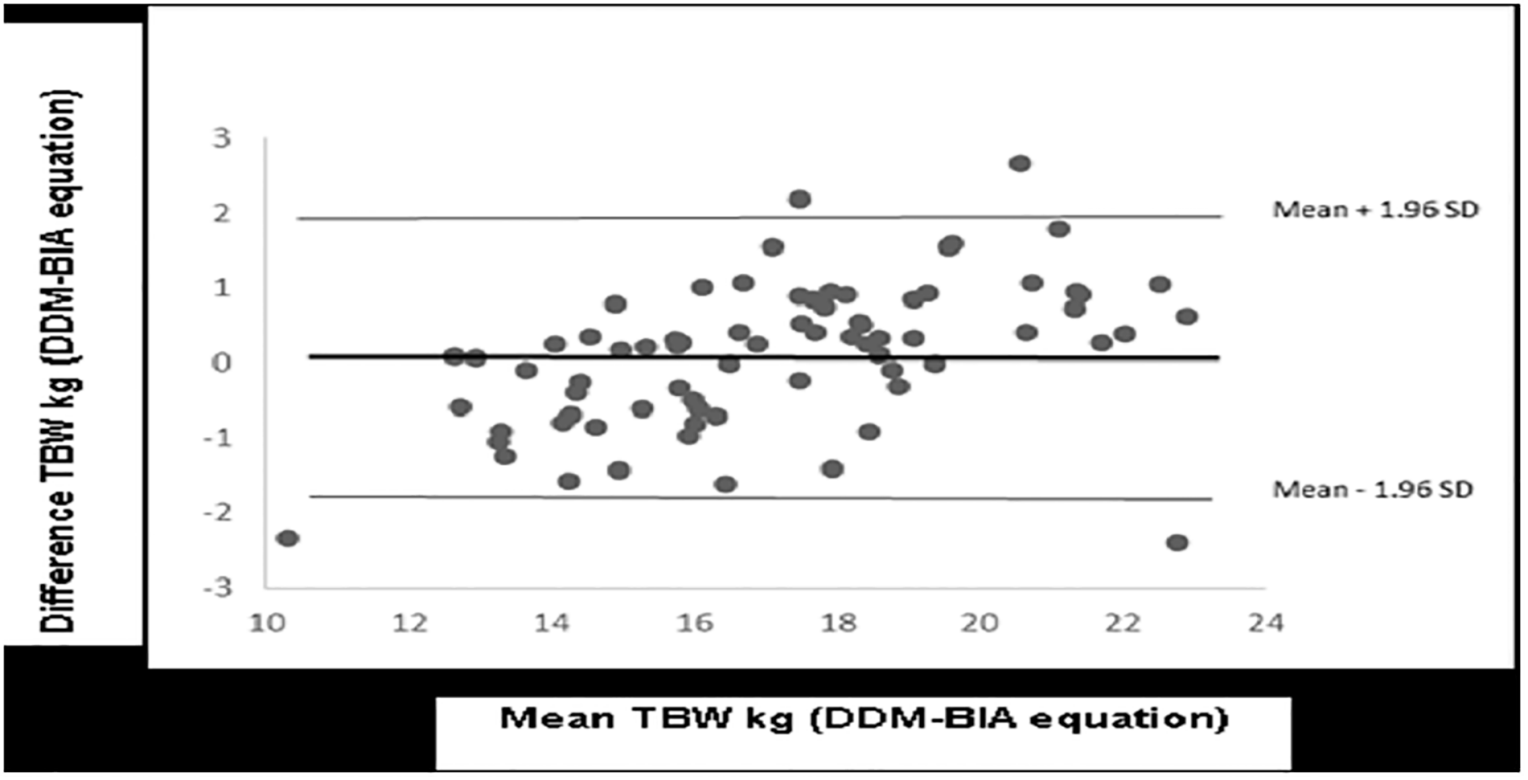
Bland and Altman plot of the difference in TBW (kg) measured by DDM and predicted by the BIA equation against their mean. The horizontal line represents the bias and the upper and lower horizontal lines indicate the 95% CI limits of agreement (±1.96 x SD) for bias.

**Fig 4:**
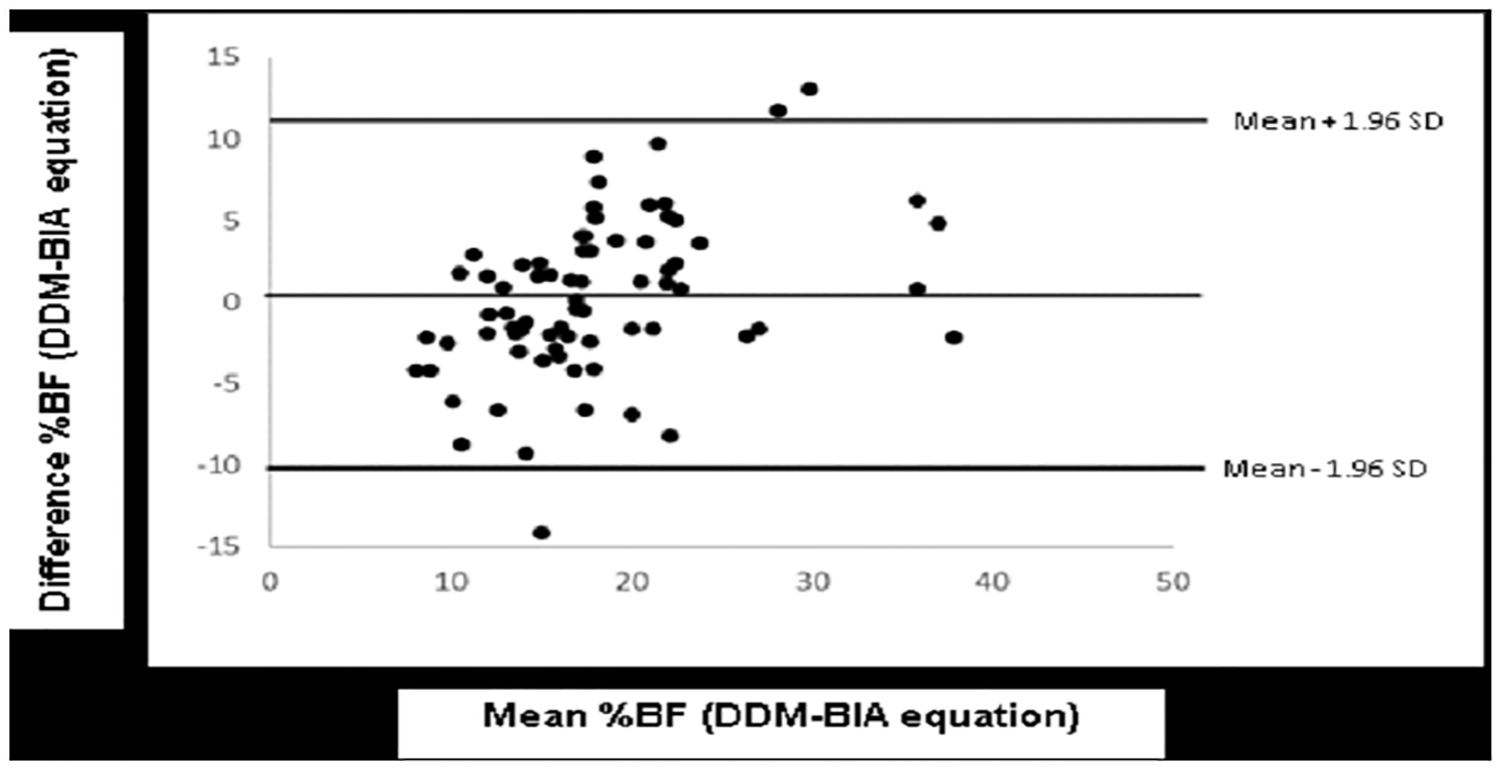
Bland and Altman plot of the differences between %BF measured by DDM and predicted by the BIA equation against their mean. The horizontal line represents the bias and the upper and lower horizontal lines indicate the 95% CI limits of agreement (±1.96 x SD) for bias.

The Bland and Altman approach used to plot the difference between the measured and predicted values against their means showed a small error and non-significant bias for TBW (0.11 kg±0.91; *P* = 0.279) and %BF (0.19% ±5.6; *P* = 0.764). The limits of agreements ranged from -1.67 to 1.89 and -10.78 to 11.17% for TBW (Fig 3) and %BF, respectively (Fig 4). Four outliers were observed in the Bland and Altman TBW plot.

## Discussion

Childhood obesity is an emerging public health problem in low and middle-income countries and highlights the need for reliable field method assessments for body composition in children. The present study was designed to develop and validate an alternative tool for the assessment of body composition (TBW and % BF) among Senegalese school-age children. Body composition measured by DDM revealed significant differences by sex. These results corroborate those obtained by Haroun and Nielsen from a sample of Asian, European and Swedish children and adolescents [15, 22]. The sexual dimorphism in body composition that mainly occurs in the prepubertal period may explain these differences [3]. Meanwhile, Ramirez found no difference in body composition between Mexican girls and boys aged 6–14 years [23]. Our study shows a different prevalence in obesity as calculated by BMI (1.9%) and DDM (11%) and highlight the limitation of BMI to accurately identify individuals with excess body fat. DDM is safe and accurate for assessing body composition, but the method is expensive and thus not well suited to large epidemiological studies. BIA is a simple and useful method for quantifying body compartments, but it is necessary to validate it against a reference. Although several validation studies exist in the paediatric population, predictive equations are generally established to be population-specific [12–16]. The BIA equations developed for the estimation of TBW and adiposity in the present study are, to our knowledge, the first to be validated among school-aged children in West African countries. The final regression model provides equations with good precision and an acceptable RMSE (TBW: R^2^ = 0.89 kg, RMSE = 0.89; %BF: R^2^ = 0.76, RMSE = 4.5%) comparable with previous equations [13, 24]. Furthermore, according to Houtkooper et al., a TBW prediction error of less than 3.0 kg for men and 2.3 kg for women would be considered very good [25]. Similarly, prediction errors observed with %BF in the present study were consistent with previous validation studies in children and adolescents ranging from 2.5% to 5.0% despite a higher adjusted correlation coefficient (R^2^) reported, which is probably due to a larger sample size in these studies [14, 26]. The %BF estimated by BIA had a lower correlation coefficient than TBW compared to reference methods because BF is an estimate based on the difference between body weight and FFM, and relative %BF is calculated accordingly [27]. Age has a relatively small effect on the predictions of both the TBW and %BF prediction equations and could be explained by the relative homogeneity of the age in our sample. These findings corroborate those reported by Nielsen et al. in a sample of 9- to 11-year-old Swedish children [15]. Our prediction equations showed an accuracy that is comparable with previously published equations of TBW [13, 24] and %BF [23–24, 28, 29]. The Bland and Altman plot [21] indicated a low and non-significant bias between BIA and the reference method. These results were consistent with those of the TBW prediction equation reported by Liu et al. from Asian schoolchildren aged 8–10 years old and de Beer among pre-school children [13, 24]. Bland and Altman TBW plot shows 4 outliers, which may be explained by the fact that when TBW measured using deuterium dilution is compared with a predicted value of TBW, it is expected that 2.5% of measurements are more than +2SD from the mean difference and 2.5% less than the mean difference (–2SD) in participants with high and low BMI, respectively (IAEA, 2010). In our study, 2.6% of measurements (2 outliers) were more than +2SD and 2.6% (2 outliers) less than -2SD was observed.

Similarly, the bias and wide limits of agreement obtained with the %BF equation were similar to those of the published equations [3, 9, 30]. By contrast, the %BF equations developed by Prins and Lee reported narrower limits of agreement in school-aged children and adolescents [10, 30]. The discrepancy between the studies may be due to the difference in age groups targeted, sample size, BIA equipment, criterion methods used and selection of independent variables. Although BIA had higher agreement with DDM, the bias and limit of agreement obtained with TBW was lower than those observed with %BF. A possible explanation for this observation lies in the nature of the BIA method, which was designed to measure TBW according to the resistance to an alternating current rather than body fat [27]. Other factors that are known to influence BIA estimates of body composition are skin temperature, variations in electrode placement, prior consumption of food or beverages and body position [27, 31]. These last three parameters were controlled in our study. The large limits of agreement of %BF plot suggest that BIA is therefore potentially useful for measuring body composition in epidemiological study, but is inaccurate for the individual. Furthermore, Our study has some limitations. We were not able to develop specific gender equations because of the low sample size, but in developed equations, sex accounted for variations in TBW and %BF. Furthermore, the equation developed in the present study should be used in similar age groups and not in older children or adolescents because of the changes that occur in the amount and distribution of FFM and FM during the growth and maturation period.

## Conclusion

The two new equations developed in this study were significantly correlated with deuterium dilution method and showed acceptable precision and accuracy. To our knowledge, this study is the first to use BIA equations for predicting TBW and %BF in school-aged children in Senegal. These equations will be a useful tool in assessing body composition and excess body fat in children. So, further cross-validation studies should be performed in a larger independent sample of African pupils.

## Abbreviations

%BF: percentage body fat
BIA: bioelectrical impedance analysis
BMI: body mass index
DDM: deuterium dilution method
TBW: total body water
